# Clinical and instrument-based assessment of balance, gait, and motor functions in pediatric cerebral palsy: A systematic review

**DOI:** 10.1017/wtc.2025.10011

**Published:** 2025-06-30

**Authors:** Dilnoza Karibzhanova, Prashant K. Jamwal, Amna Riaz Khawaja, Zaidagul Kystaubayeva, Akim Kapsalyamov, Meiram Shakenov, Sunil Agrawal

**Affiliations:** 1 School of Medicine, Nazarbayev University, Astana, Kazakhstan; 2 School of Engineering and Digital Sciences, Nazarbayev University, Astana, Kazakhstan; 3 Department of Family Medicine, Astana Medical University, Astana, Kazakhstan; 4 Faculty of Engineering and Mathematics, Hochschule Bielefeld University of Applied Sciences, Bielefeld, Germany; 5 Department of Rehabilitation, National Center for Children’s Rehabilitation, Astana, Kazakhstan; 6 Department of Mechanical Engineering and the Department of Rehabilitation and Regenerative Medicine, Columbia University, New York, NY, USA

**Keywords:** feedback devices, biomechanics, embedded electronics, performance characterisation

## Abstract

Specialists globally employ various clinical scales and instruments to assess balance, gait, and motor functions in children with cerebral palsy (CP). Selecting appropriate assessment tools is essential for planning studies, developing effective treatment strategies, and tracking clinical outcomes. Given the diversity in assessment needs – whether evaluating dynamic, functional, or static balance – there is a need to identify the most suitable tools for each aspect. Therefore, the primary objective of this review is to critically analyze current clinical and instrument-based assessment methods in the literature to determine the most effective approaches for pediatric CP. This systematic review retrieved 1,812 papers, of which only 23 met the inclusion criteria and presented assessment methods for evaluating balance and motor functions in pediatric CP. These methods were further organized into clinical and instrument-based assessment groups. Among clinical examinations, the Pediatric Balance Scale and Gross Motor Function Measures were considered gold standards and featured in eight studies. In contrast, postural sway measured with the Biodex Balance System, Gait Stability Indices from the GAITRite system, and EMG sensing were the predominant instrument-based observations. Despite this variety, a consensus on the best assessment methods remains lacking. This review highlights the potential of integrating AI-driven metrics that combine clinical and instrument-based data to enhance precision and individualized care. Future research should focus on creating integrated, individualized profiles to better capture the unique capabilities of children with CP, enabling more personalized and effective intervention strategies.

## 1. Introduction

Cerebral palsy (CP) is a group of disorders caused by abnormalities or injuries to the brain during early development, which results in motor impairments and consequent gait- and balance-related disorders. It is the leading cause of physical disabilities in children, affecting approximately 17 million individuals worldwide (Cortés-Pérez et al., 2022) and adversely impacting their daily activities and self-care.

Children with CP often experience poor balance reactions and significant impairments in dynamic balance, which can affect their safety and limit mobility (Niiler, 2020). Balance issues in CP are linked to various deficits, including spasticity, altered motor control, limited joint range of motion, muscle weakness, and impaired postural responses (Tomita et al., 2024). Balance is typically defined as the ability to maintain the body’s center of mass (CoM) within the base of support (BoS), which is the area around and between the feet (Phuaklikhit and Junsri, 2023). The center of pressure (CoP) is another key indicator, representing the origin of the ground reaction vector (Pavão et al., 2013). Although many studies assess CoP using pressure sensors, CoM and BoS are also critical for evaluating balance and fall risk (Ishii et al., n.d.). Maintaining balance involves constant adjustments of the CoP to control the CoM’s position within the BoS, especially during dynamic activities like walking or turning (Jeon et al., 2021).

Three main categories of human activity are associated with the regulation of balance: (1) maintaining a static position, such as sitting or standing; (2) Voluntary movement or activity that tends to move the body, which means more intense interaction with the environment, for example, walking and running; (3) Reaction to an external incident or activities including switching between postures (Antar et al., 2019; Ahad et al., 2021; Yu et al., 2022). Balance can be categorized into static, dynamic, and functional balance. Static balance refers to maintaining equilibrium while stationary, whereas dynamic balance involves maintaining stability during movement (Niiler, 2020). Dynamic balance is the ability to maintain the CoM within the BoS during various movements such as walking, turning, or other leg movements (Rizzato et al., 2023). However, the BoS is poorly determined during movement, when one or both feet are not on the ground, and therefore, alternate approaches are required for the assessment of dynamic balance (Niiler, 2020). Functional balance encompasses the ability to maintain body orientation when performing transitional movements such as sit-to-stand or walking upstairs, integrating both static and dynamic components (Özal et al., 2023). The ability to maintain the body’s center of mass within a base of support while moving quickly in many directions is known as functional or postural balance (Alonso et al., 2014). Therefore, functional balance can also be considered as a combination of static and dynamic balance. Activities with postural changes can be further divided into four types: static to static postural transition (stand to sit, lie to sit, etc.), static to dynamic (still to walk), dynamic to static (walk to still) and dynamic to dynamic (walk to jog, jog to run, etc.) (Yu et al., 2022). The challenge for clinicians lies in selecting appropriate assessment tools (AT) that accurately measure different aspects of balance.

Therapists and researchers use clinical scales and advanced instruments to assess balance in CP (Pérez-López et al., 2023). Clinical scales monitor impairments and treatment outcomes, while instrumental tools offer precise measurements of physical parameters (Giannoni and Zerbino, 2022). Selecting the right assessment tools is crucial for creating effective treatment plans, tracking progress, and making informed decisions (Sibley et al., 2013). However, it is also important for practitioners to fully understand the mechanisms of balance and postural control before they choose a clinical scale or instrument. For example, a study by Stergiou et al. focuses on interventions aimed at improving balance (Stergiou et al., 2024). Although the title of the article lists exercises aimed at improving static and dynamic balance, the content mainly discusses the use of the Pediatric Balance Scale (PBS) to assess functional balance. Such gaps indicate that researchers may have difficulty accurately classifying balance and choosing appropriate assessment tools. Choosing the right assessment tools is essential in the clinical management of CP, and they should be reliable and valid for the target population, easy to use and understand, and flexible enough to adapt to changes (Saether et al., 2013). Using the right tools helps clinicians and researchers develop personalized treatments, track progress, and make informed decisions (Pashmdarfard and Araghi, 2022).

Although several systematic reviews of assessment tools for assessing balance in CP have been published (Niiler, 2020; Saether et al., 2013; Sibley et al., 2013), these studies focus mostly on clinical methods and questionnaires, excluding instrumental methods and wearable devices. Other studies, which focus on Inertial Measurement Units (IMUs), Electromyography (EMG), pressure platforms, and force plates, study them individually and do not provide information about other assessment devices. The review by Caldas et al. presents a comprehensive analysis of gait assessment methods based on IMUs and adaptive algorithms and demonstrates their high accuracy in measuring spatiotemporal parameters such as step length, rhythm, and asymmetry (Caldas et al., 2017). However, the study does not consider the possibility of using these technologies to assess balance, which is an equally important aspect of mobility in CP. Pavão et al. focus on methods for studying posture control, such as kinematic and dynamic analysis, dynamometry, and EMG, but do not consider the potential of wearable sensors and other tools for assessing balance and gait (Pavão et al., 2013). The authors note that the analysis of postural control while performing functional tasks is important for understanding balance in everyday life. A recent systematic review and meta-analysis conducted by Mao et al. provides valuable data on sensor-based interventions to improve gait and balance in older adults, focusing on clinical assessment results (Mao et al., 2024). However, the study does not consider their applicability in the pediatric population, especially in CP, and does not address the issue of instrumental assessment tools. With the development of technology, it is crucial to consider the instrumental tools that can provide additional insights into balance assessment. Therefore, this study aims to address this gap by identifying both clinical scales and instrumental tools, as well as specific parameters used in assessing children with CP that have been employed in recent years. State-of-the-art balance assessment tools are revealed, which can be useful for clinicians and researchers in evaluating balance capabilities among children with CP.

The other most significant challenges faced by children with CP are gait abnormalities, which severely impair their ability to walk and participate in daily activities. Gait training is a crucial aspect of rehabilitation for these children as it aims to improve their walking ability, increase independence, and enhance their overall quality of life. Gait training for children with CP focuses on improving muscle strength, coordination, and balance, enabling them to develop a more efficient walking pattern. This training also helps in mitigating the risk of secondary complications such as joint deformities and pain. Early intervention with gait training is particularly important as it can take advantage of the plasticity of the developing brain, potentially leading to better long-term outcomes. Gait spatio-temporal parameters are critical metrics used to evaluate and monitor the walking patterns of children with CP (Franjoine et al., 2003; Lim, 2015). These parameters provide valuable insights into the effectiveness of gait training and the progression of the disorder. Assessing gait in children with CP is essential for diagnosing gait abnormalities, planning interventions, and evaluating the effectiveness of treatments. Through a combination of clinical observations (Sarathy et al., 2019) and instrument-based measurements (Shrader et al., 2021), healthcare professionals can effectively assess gait abnormalities and implement targeted treatments. During clinical observation, initial insight into the deviations in spatio-temporal parameters such as step length, stride length, cadence, Stance and Swing Phases, and so forth are observed. Other clinical assessments include Six-Minute Walk Test (6MWT), Timed Up and Go (TUG) Test, Ten-Meter Walk Test (10MWT), and so forth (Franjoine et al., 2003; Lim, 2015). Instrument-based methods employ markerless and 3D motion capture systems, pressure measurement platforms, force plates, IMUs, EMG systems, and so forth These instrument-based methods provide quantifiable gait data, joint ranges of motions (ROMs), muscle activations, and so forth, which are critical for diagnosing gait abnormalities, planning rehabilitation, and monitoring progress in individuals with gait impairments.

Similarly, motor function assessment is also crucial for children with CP as it helps in understanding the extent of motor impairments, guiding effective intervention, and monitoring progress over time. Children with CP often face challenges such as muscle spasticity, weakness, poor coordination, and so forth that significantly affect their ability to perform everyday activities. Key motor function parameters for children with CP include gross motor skills like walking and standing, fine motor skills such as hand-eye coordination, and muscle tone, strength, and endurance (Wu et al., 2024). These parameters provide insights into the child’s ability to move, maintain posture, and manipulate objects. Various clinical and instrument-based assessment methods are used to evaluate motor functions in children with CP. Clinical scales like the Gross Motor Function Measure (GMFM) and the Manual Ability Classification System (MACS) are commonly employed to assess motor capabilities (Ciccodicola et al., 2024). Additionally, instrumental tools such as motion capture systems, dynamometers, and EMG are used to quantify movement patterns and muscle activity (Chen et al., 2020). These assessments are vital for tracking the effectiveness of interventions and adapting therapy to the child’s evolving needs.

## 2. Materials and methods

### 2.1. Protocol and registration

This systematic review was registered on the International Prospective Register of Systematic Review (PROSPERO) with the ID number CRD42024602025, and the PRISMA guideline was followed to ensure a comprehensive literature review.

### 2.2. Search strategy

**Figure 1.**
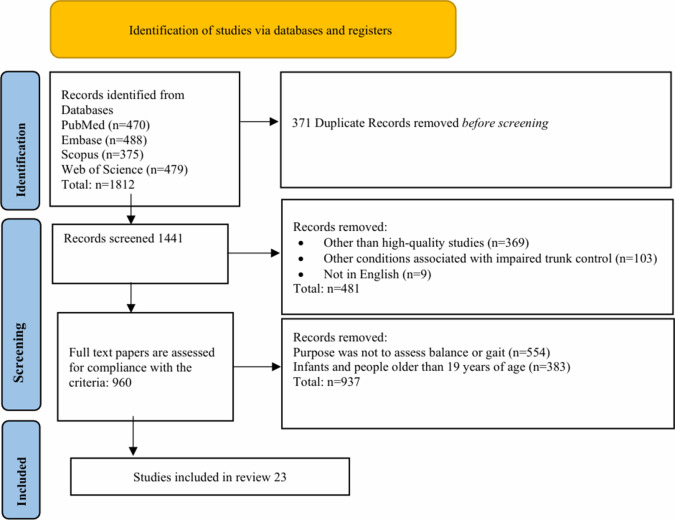
PRISMA flowchart on the selection for papers.

We conducted a comprehensive literature search for studies published up to 2024, utilizing keywords such as “balance and gait assessment,” “cerebral palsy assessment,” and “instrument-based CP assessment,” among others (see Table 1 for the complete list of keywords). The search was carried out across multiple databases, including PubMed, Embase, Scopus, and Web of Science, to ensure a thorough and inclusive review of relevant studies. We conducted two separate searches: one for clinical assessments and another for instrument-based assessments, focusing on specific metrics for analyzing balance, gait, and motor functions to emphasize the necessity of the integration of traditional clinical scales with modern instrument-based metrics. Figure 1 illustrates the manuscript selection process, focusing on studies that employed balance and gait assessment tools.

**Table 1.** Searching strategy for clinical assessments (green color) and instrument-based assessments (blue color)

Database	Keywords	Hits
Pubmed	((“cerebral palsy”[MeSH Terms] OR (“cerebral”[All Fields] AND “palsy”[All Fields]) OR “cerebral palsy”[All Fields]) AND (“balance”[All Fields] OR “balanced”[All Fields] OR “balances”[All Fields] OR “balancing”[All Fields]) AND (“gait”[MeSH Terms] OR “gait”[All Fields]) AND (“assess”[All Fields] OR “assessed”[All Fields] OR “assessement”[All Fields] OR “assesses”[All Fields] OR “assessing”[All Fields] OR “assessment”[All Fields] OR “assessment s”[All Fields] OR “assessments”[All Fields])) balance: “balance”[All Fields] OR “balanced”[All Fields] OR “balances”[All Fields] OR “balancing”[All Fields] gait: “gait”[MeSH Terms] OR “gait”[All Fields] assessment: “assess”[All Fields] OR “assessed”[All Fields] OR “assessement”[All Fields] OR “assesses”[All Fields] OR “assessing”[All Fields] OR “assessment”[All Fields] OR “assessment’s”[All Fields] OR “assessments”[All Fields]	192
	“instrument-based”[All Fields] AND (“assess”[All Fields] OR “assessed”[All Fields] OR “assessement”[All Fields] OR “assesses”[All Fields] OR “assessing”[All Fields] OR “assessment”[All Fields] OR “assessment s”[All Fields] OR “assessments”[All Fields]) AND (“balance”[All Fields] OR “balanced”[All Fields] OR “balances”[All Fields] OR “balancing”[All Fields]) AND (“gait”[MeSH Terms] OR “gait”[All Fields]) cerebral palsy”[MeSH Terms] OR (“cerebral”[All Fields] AND “palsy”[All Fields]) OR “cerebral palsy”[All Fields] functional balance: “functional”[All Fields] OR “functional’s”[All Fields] OR “functionalities”[All Fields] OR “functionality”[All Fields] OR “function”[All Fields] OR “physiology”[MeSH Terms] dynamic balance: “dynamer”[All Fields] OR “dynamers”[All Fields] OR “dynamic”[All Fields] OR “dynamical”[All Fields] OR “dynamically”[All Fields] OR “dynamicity”[All Fields] OR “dynamics”[All Fields] OR “dynamism”[All Fields] OR “dynamisms”[All Fields] “gait”[MeSH Terms] OR “gait”[All Fields]) AND (“electromyography”[MeSH Terms] OR “electromyography”[All Fields] OR “emg”[All Fields]) AND (“cerebral palsy”[MeSH Terms] OR (“cerebral”[All Fields] AND “palsy”[All Fields]) OR “cerebral palsy”[All Fields] “Markerless”[All Fields] AND (“motion capture”[MeSH Terms] OR (“motion”[All Fields] AND “capture”[All Fields]) OR “motion capture”[All Fields]) “motion capture”[MeSH Terms] OR (“motion”[All Fields] AND “capture”[All Fields]) OR “motion capture”[All Fields]	278
Embase	“balance gait assessment cerebral palsy” OR ((“balance”/exp OR balance) AND (“gait”/exp OR gait) AND (“assessment”/exp OR assessment) AND cerebral AND (“palsy”/exp OR palsy)) “cerebral balance gait clinical assessment” OR (cerebral AND (“palsy”/exp OR palsy) AND (“balance”/exp OR balance) AND (“gait”/exp OR gait) AND (“clinical”/exp OR clinical) AND (“assessment”/exp OR assessment))	241
	“balance gait instrumental assessment” OR ((“balance”/exp OR balance) AND (“gait”/exp OR gait) AND instrumental AND (“assessment”/exp OR assessment) AND electromyography AND (“assessment”/exp OR assessment) OR emg AND motion capture system AND markerless motion capture OR motion capture AND marker-based analysis OR gait analysis	247
Scopus	balance AND gait AND assessment AND cerebral AND palsy AND instrument-based AND assessment AND tools AND (LIMIT-TO (LANGUAGE, “English”)) balance AND instrument-based AND assessment AND tools AND balance AND 6-minute AND walk AND test, AND timed AND up AND go AND test, AND 10-meter AND walk AND test AND cerebral AND palsy AND (LIMIT-TO (LANGUAGE, “English”))	272
	“balance and gait assessment” OR “instrument-based assessment tools” OR “cerebral palsy assessment” OR “balance assessment cerebral palsy” AND (LIMIT-TO (LANGUAGE, “English”)) “markerles motion capture system” OR “marker-based analysis” OR “gait analysis” OR “markerless gait analysis”	103
Web of Science	(balance assessment cerebral palsy) and Selective Motor Control (OR – Search within topic) and Gross Motor Function (OR – Search within topic) and Spastic Cerebral Palsy (OR – Search within topic) and Trunk Control (OR – Search within topic) and Cerebral Palsy (OR – Search within topic) and Spastic Diplegia (OR – Search within topic) (balance and gait assessment) and Functional Gait Assessment (OR – Search within topic) and Postural Balance (OR – Search within topic) and Fall Risk (OR – Search within topic)	386	
	instrumental-based assessment (All Fields) and Dynamic Gait Index (OR – Search within topic) and Balance Assessment (OR – Search within topic) and Balance (OR – Search within topic) and Gait (OR – Search within topic) and Gait Analysis (OR – Search within topic) and Postural Control (OR – Search within topic) emg (All Fields) and cerebral palsy (All Fields) and balance (All Fields) and motion capture system (All Fields) and markerless motion capture (OR – Search within topic) and motion capture (All Fields) and marker-based analysis (OR – Search within topic) and gait analysis (All Fields)	93	

### 2.3. Inclusion/exclusion criteria

#### 2.3.1. Inclusion Criteria

This review focused on articles that:

- Were randomized controlled trials and high-quality observational studies (e.g., cohort or cross-sectional studies) published between 2019 and 2024, capturing the latest five-year period to form a recent and extensive evidence base.
- Assessed balance and gait in children with any level of Gross Motor Function Classification System (GMFCS) and type of CP, between 2 and 19 years old.
- Utilized clinically reliable and widely used measurement tools to assess balance and gait.
- Were published in English.

#### 2.3.2. Exclusion Criteria

Articles were excluded during full-text review if:

- The primary purpose of the measurement tool was not to assess balance or gait.
- Infants and people older than 19 years of age, as well as children with other conditions associated with impaired trunk control, were used.
- Review papers, pilot studies, conference abstracts, thematic reports, and non-peer-reviewed papers had limited methodological rigor.
- Papers were published in languages other than English.

### 2.4. Study selection

Three reviewers (D.K. and A.R.K., and A.K.) independently screened the articles that were found in all databases. After removing the duplicates, reviewers screened the titles and abstracts to select eligible articles using predefined inclusion and exclusion criteria, after which the full texts were uploaded. Any disagreements were resolved through consultations with the fourth reviewer (P.K.J.).

The articles were read in full, and data from the articles were extracted by the two reviewers (Z.K. and M.Sh.). The authors and year of publications, experimental design, population, objectives, Clinical Assessment Tools, and Instrumental Assessment Tools were registered in Table 2. Any disagreement was discussed with a third reviewer (S.K.A.).

**Table 2.** A list of clinical and instrumental tools used for balance, gait, and motor functions assessment

Author/Year	Experimental design	Duration	Population				Objective	Clinical Assessment Tools	Instrumental Assessment Tools
			Sample size (n)	Age(years)	GMFCS	Type of CP			
Ali et al. (2019)	A randomized controlled trial	12 weeks	72	5–8	–	Hemiplegic and diplegic	Static balance	–	Biodex Balance System (Biodex Medical System, NY, USA)
Elnaggar et al. (2023)	A randomized clinical study	12 weeks	52	10–16	I or II	Unilateral CP	Dynamic balance	6MWT, TUDS, and the Ten-meter Shuttle Run Test (10mSRT)	The portable GAITRite system (GAITRite, CIR System, Clifton, NJ, USA) and Biodex balance system (Biodex Medical System, NY, USA)
Cho and Lee (2020)	A randomized controlled trial	6 weeks	28	6–13	I–III	Diplegia CP	Motor function and dynamic balance	The Functional Reach Test (FRT), GMFM–88.	Baseline Absolute Axis digital goniometer
Rapson et al. (2023)	A cross-sectional study	–	30	8–18	I-III	Spastic unilateral and bilateral	Dynamic balance	Goniometry and the Modified Tardieu Scale, GMFM, and Quality Functional Measure (QFM)	Portable force plate (Kistler™ 9286BA UK), CODAmotion™ (Leicestershire, UK) electronic markers, and a hand-held dynamometer (Lafayette, USA)
Hsieh (2020)	A randomized controlled trial	12 weeks	56	6–10	I–III	Any type of CP	Dynamic balance	Pediatric Balance Scale (PBS) and the 2-min walk test (2MWT)	Portable force platform The Zebris FDM System (Zebris Medical GmbH, Isny, Germany)
Stergiou et al. (2024)	A randomized controlled trial	12 weeks	27	–	I–II	–	Dynamic and static balance	PBS, Wechsler Intelligence Scale for Children (3rd edition), and GMFM	Foot pressure-sensitive walkway-Win-Track (Medicapteurs France SAS, VALIDATION)
Elnaggar et al. (2024)	A randomized controlled trial	12 weeks	81	12–18	I–II	Spastic unilateral CP	Functional balance	Community Balance and Mobility Scale (CB&M); Functional Walking Test (FWT); Timed Up and Down Stair test (TUDS)	Biodex balance system (Biodex Medical System, Shirley, NY, USA)
An et al. (2024)	A randomized, single-blinded trial	4 weeks	30	10–19	II–III	Hemiplegic CP	Functional, dynamic, and static balance, gait function	6 MWT, BBS, Functional Ambulation Category (FAC), and Modified Barthel index (MBI)	NA
Jha et al. (2021)	A randomized controlled trial	6 weeks	38	6–12	II–III	Bilateral spastic	Functional balance	PBS, Kids-Mini-Balance Evaluation System Test (Kids-Mini-BESTest), GMFM–88, and Wee-Functional Independence Measure (WeeFIM)	–
Mohammed et al. (2023)	A randomized controlled trial	12 weeks	54	6–9	–	Hemiplegic CP	Functional balance and gait stability	Selective Control Assessment of Lower Extremity Scale (SCALE) and The Pediatric Balance Scale (PBS)	–
Khalil et al. (2023)	A Randomized Clinical Trial	6 months	39	5–10	I–II	Hemiplegic CP	Functional balance and gait stability	The Pediatric Balance Scale	The GAITRite System (CIR Industries, Clifton, NJ, USA)
Bilek and Tekin (2021)	A Randomized Controlled Trial	8 weeks	32	7–13	I–II	Unilateral CP	Static and dynamic balance	Trunk Control Measurement Scale, Trunk Impairment Scale, Pediatric Balance Scale (PBS), and Timed Up and Go test (TUG)	–
Chaovalit et al. (2021)	A single-blind randomized controlled trial	6 weeks	38	4–12	III and IV	Any type of CP	Dynamic balance	WeeFIM, Five Times Sit-to-Stand Test (FTSST), and Modified Caregiver Strain Index (MCSI).	–
Kara et al. (2019)	A randomized controlled trial	12 weeks	43	7–16	level I	Unilateral CP	Motor function and gait stability	The gross motor function classification system expanded and revised (GMFCS-E&R) and manual ability were classified using the MACS and the muscle power sprint test, short-term muscle power, TUG, and 1MWT	Power Track II Commander (JTECH Medical, Salt Lake City, Utah).
Rastgar Koutenaei et al. (2023)	Single-blind, randomized superiority trial.	5 weeks	30	6–12	I–III	Spastic CP	Static balance and gait stability	The Trunk Control Measurement Scale (TCMS), abdominal muscle thickness, Pediatric Balance Scale (PBS), standing and walking sections of GMFM–88, and mobility section of the Pediatric Evaluation of Disability Inventory (PEDI)	Diagnostic ultrasound imaging unit (SONOACE R7, SAMSUNG MEDISON CO. LTD, Korea)
Elshafey et al. (2022)	A randomized controlled trial	2 months	45	5–9	IV	Cerebellar ataxic CP	Static balance	The Balance Error Scoring Systems scale, Bruininks–Oseretsky tests of motor proficiency, the Scale for the Assessment and Rating of Ataxia (SARA)	HUMAC balance system scores (CSMi, Stoughton, MA, USA)
Chinniah et al. (2020)	A randomized controlled trial	12 weeks	30	2–4	I–III	Spastic CP	Motor function	Sitting motor function was assessed by GMFM–88 (sitting dimension B).	–
Yun et al. (2023)	A cross-sectional study		36	5–16	I–II	Spastic bilateral	Motor function, gait stability, and functional balance	Selective Control Assessment of the Lower Extremity (SCALE), GMFM–88 D&E, PBS, the Ten-Meter Walk Test (10MWT), and TUG	Video-based observational gait analysis tools – the Edinburgh Visual Gait Score (EVGS)
Fu et al. (2022)	A randomized controlled trial	12 weeks	60	6–11	II–III	Spastic	Gait stability	The Modified Ashworth Scale, the Gross Motor Function Measure (GMFM) dimensions E and D, and Six-Minute Walk Test (6 MWT)	Electromyography (EMG)
Hegazy et al. (2020)	A single-blind randomized controlled trial	3 months	30	4–6	–	Spastic hemiplegic	Gait stability	–	EMG and gait analysis were assessed using Qualisys motion capture system (ProreflexMCU500Hz, QualisysMedicalAB, 257 Gothenburg, Sweden)
Wishaupt et al. (2024)	A cross-sectional study	Single session	45	7–16	I–III	Spastic	Gait stability	–	The markerless camera system (7 Blackfly S USB3 cameras, Teledyne FLIR LLC, Wilsonville Oregon, USA), and the marker-based camera system (4 Vicon Vantage V5 cameras + 8 Vicon T40S, Vicon Motion Systems Ltd., Oxford, United Kingdom); Theia3D markerless system (Theia Markerless Inc., Kingston, ON, Canada) and a marker-based system (Human Body Model (HBM))
Rasmussen et al. (2019)	A prospective, single-blind, parallel-group, randomized controlled trial	52 weeks	60	5–8	I–II	Spastic	Gait stability	Functional mobility scale, one-minute walk test	Video recording and three-dimensional kinematics and kinetics by an eight-camera Vicon T40 system (Vicon, Oxford, UK) and two force-plates (OR6–7–1000; AMTI, Watertown, MA, USA) Gait Deviation Index (GDI)
Flux et al. (2020)	A cross-sectional study	Single session	25	8–15	I–II	spastic	Gait stability	–	Plug-in-Gait (PiG), the calibrated anatomical system technique (CAST), and the human body model (HBM)

**Table 3.** Summary of methods of assessing balance and gait

	Static balance	Dynamic balance	Functional balance	Gait
Clinical test	BESS TIS (the static subscale)	6 MWT 10MWT FWT 10mSRT FAC FTSST TIS (dynamic subscale)	GMFM TUGT TUDT BBS or PBS CB&M TCMS FRT QFM MBI	GMFM 6 MWT 10MWT FWT 10mSRT FAC TUGT
Instrumental assessment	Accelerometer IMU EMG Pressure platforms HUMAC balance system	Accelerometer IMU EMG The GAITRite System	Accelerometer IMU EMG Pressure platforms The GAITRite System	Accelerometer IMU EMG The GAITRite System Video-Based Gait Analysis Motion Capture systems (markerless and marker-based)
Measured parameters	CoP CoM the sway area, path, and sway velocity acceleration jerk frequency	CoM Spatiotemporal parameters	Combination of static and dynamic balance parameters	CoM Temporal parameters (stride time, velocity, and single or double support) Spatial parameters (step length, stride length, and distance)

### 2.5. Data extraction and quality assessment

In total, 20 articles utilizing clinical measures and instrumental tools for balance and gait assessment were reviewed. Details about the study and characteristics of the balance and gait assessment tools from these articles were systematically organized into a predefined table based on the CanChild Outcome Measures Rating Form. The methodological quality of the studies was assessed following the Standards for Selecting Health Measures (COSMIN), which outlines a ten-step procedure for conducting systematic reviews. Additionally, we assessed the quality and feasibility of each study according to COSMIN guidelines specific to systematic reviews of patient-reported outcome measures (PROMs). This involved identifying the measurement properties evaluated in each article and extracting data on PROM performance. Inconsistencies among subgroup results were noted and summarized accordingly.

## 3. Results

This section provides a brief overview of the results from the literature analysis devoted to the research goal of critically evaluating clinical and instrument-based methods for assessing balance, gait, and motor functions in pediatric CP. A total of 458 search results through PubMed, 471 through Embase, 369 through Scopus, and 473 articles through Web of Science articles were found related to the various clinical and instrument-based tools used to assess patients with CP.

**Figure 2.**
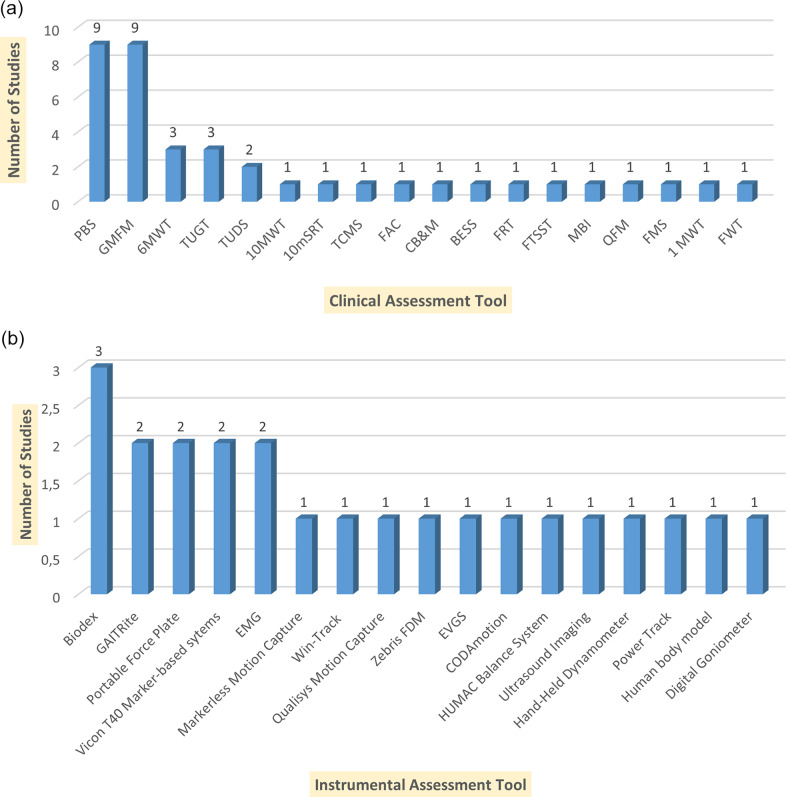
Summarizing the clinical assessment tools and the number of studies in which each tool was utilized. (a) Clinical Assessment Tools and Their Usage Across Studies. Pediatric Balance Scale (PBS); Gross Motor Function Measure (GMFM); Six-Minute Walk Test (6MWT); Ten-Meter Walk Test (10MWT); Ten-meter Shuttle Run Test (10mSRT) Timed Up and Go Test (TUGT); Timed Up and Down Stairs (TUDS); Functional Ambulation Category (FAC); Trunk Control Measurement Scale (TCMS); Community Balance and Mobility Scale (CB&M); Balance Error Scoring System (BESS); Functional Reach Test (FRT); Functional Mobility Scale (FMS); One-Minute Walk Test (1MWT); Five Times Sit-to-Stand Test (FTSST); Functional Walking Test (FWT). (b) Instrumental Assessment Tools and Their Usage Across Studies.

Figure 2a shows the most common clinical assessments, emphasizing their importance in assessing the functional capabilities of patients with CP. The most commonly used were the PBS and the General Motor Function Measurement Scale (GMFMS), each being reported in nine studies. These tools are widely known for their effectiveness in assessing balance, mobility, and motor activity, making them valuable both in clinical practice and in scientific research. A 6MWT (three studies) and TUG test (three studies) were also often used to assess endurance and functional mobility in children with CP. Instrumental assessment methods include advanced technologies that allow objective and accurate measurement of balance, gait, and motor functions in patients with CP. The most frequently used systems were Biodex Balance (three studies) and GAITRite (two studies), which emphasize their importance for quantitative analysis of gait and posture stability (Figure 2b). Additionally, power platforms, EMG, and motion capture systems are used, which play a key role in analyzing muscle activity, pressure distribution, and dynamic balance control.

The study designs included 14 randomized controlled trials, five single-blind or superiority trials, and four cross-sectional studies. The included studies varied in duration, sample size, participant age, and CP classifications. Study durations ranged from 4 to 6 weeks (six studies) from 8 to 12 weeks (10 studies), with five studies extending beyond two months, and two studies were single sessions. Sample sizes also varied widely, with the smallest study involving 27 participants (Stergiou et al., 2024) and the largest, including 81 participants (Elnaggar et al., 2024). The participant age range spanned from as young as 2 years old (Chinniah et al., 2020) to 19 years old (An et al., 2024). CP classifications covered various subtypes, with spastic CP examined in 11 studies, hemiplegic CP in six, diplegic CP in three, and unilateral CP in five. Additionally, studies assessed different severity levels using the GMFCS, with 12 studies focusing on GMFCS Levels I–III and two studies on Levels III–IV.

Six of the selected studies examined the ability of participants with CP to maintain static balance. Ali et al. used the Biodex Balance system to measure the static stability of posture in children with hemiplegic and diplegic CP for 12 weeks (Ali et al., 2019). Stergiou et al. introduced a foot pressure–sensitive track (Win-Track) to analyze the stance (Stergiou et al., 2024). Rastgar Koutenai et al. assessed static balance using the standing section of GMFM-88 (Rastgar Koutenaei et al., 2023). Elshafey et al. focused on children with cerebellar ataxia, using the Balance Error Assessment system and the HUMAC balance system to assess static balance (Elshafey et al., 2022). In addition, Bilek and Tekin performed the Trunk Impairment Scale (TIS) to assess static and dynamic balance in a sitting position (Bilek and Tekin, 2021). The TIS includes an assessment of such abilities as maintaining balance in a cross-legged position and performing body movements, with a score range from 0 to 23 (Verheyden and Kersten, 2010). These diverse methodologies highlight the variety of approaches used to assess static equilibrium in people with CP.

The choice of diagnostic tools should align with the specific interventions and balance parameters under consideration. For instance, static balance assessments often employ the Balance Error Scoring System (BESS). This tool evaluates static balance during three different stances: two-legged, single-legged, and tandem. These assessments are conducted on both hard and foam surfaces for ground perturbation with the individual’s eyes closed (Lowe et al., 2022). However, children with CP often struggle to maintain balance, especially when standing on one leg. Those with more severe conditions (GMFCS level 3 and above and who need an assistive device or wheelchair for mobility) may find it challenging to remain stable even with their eyes open. To accommodate these challenges, some research protocols modify the assessment method by using the BESS to evaluate postural stability on both solid ground and a foam block while the children stand on both feet (further details in Table 2) (Elshafey et al., 2022). Nevertheless, clinical assessments tend to be subjective and can provide only initial insight into the potential mechanisms underlying the imbalance, highlighting the need for a quantitative approach using precise instruments to evaluate standing balance.

While standing, a person must be able to control the vertical projection of the center of mass within the base of support in the anterior–posterior (AP, front-to-back) and medial–lateral (ML, side-to-side) directions to achieve postural stability. Measuring body sway using an accelerometer around the waist can capture these CoM movements as the waist is closer to the body’s CoM (Alqahtani et al., 2020). Accelerometers also provide quantitative postural fluctuations while standing and also distinguish between test conditions that require various levels of balance (Alqahtani et al., 2020). Similarly, IMUs, motion capture systems, and force plates are used as an alternative to measure balance biomarkers based on CoM and CoP in children with CP (Noamani et al., 2023). For precise measurement, pressure platforms with embedded pressure sensors are used to measure the pattern of CОP fluctuations in the anteroposterior and mediolateral directions, such as velocity and sway area (Hsieh, 2020; Reina et al., 2022). In Hsieh’s 2019 study, static postural control parameters were obtained using the Zebris FDM System, a portable force platform (see Table 2 for more details). Subsequently, the following CoP kinematics were measured: (a) sway path, which is the total path traveled by the CoP under the foot; (b) sway area of an ellipse enclosing 95% of CoP movement; and (c) the sway velocity, which is the total CoP displacement divided by time in anteroposterior sway and lateral sway (Hsieh, 2020). Stergiou *et al*. used a foot pressure–sensitive walkway called Win-Track, manufactured by Medicapteurs France SAS, to assess static balance (see Table 2 for more details) (Stergiou et al., 2024). This instrument analyzes static, postural, and gait analysis in stance position and movement (Medicapteurs, WIN-TRACK, 2017).

Wider use of the CoM and CoP measures in assessing standing balance requires an understanding of the aspects of postural control they reflect, as well as methods for interpreting them. Such an instrument is a wearable IMU that integrates a three-axis accelerometer, gyroscope, and magnetometer in one device. IMUs have been successfully used as a reliable alternative to obtain accurate and sensitive measures of static balance with proper positioning and calibration applied in various populations (Noamani et al., 2023). When analyzing static balance using wearable IMUs, important metrics are acceleration, jerk, sway area, velocity, and frequency, which should be calculated in both AP and ML directions (Hansen et al., 2021; Scataglini et al., 2021). In Rapson et al.’s 2023 study, children stood on a portable force plate (Kistler™ 9286BA UK) (see Table 2 for more details) (Kistler, n.d.). Elshafey et al. used HUMAC balance system scores that combine a balance board with advanced training software, providing a comprehensive computerized solution for balance assessment and training (Elshafey et al., 2022). Codamotion^®^ is a commercial motion capture system that is used to measure coordinates in 3D and in real-time, with fully automatic marker labeling for gait analysis of children with CP (CODAmotion, CODAmotion, n.d.). In another study, a stepping task to laterally and medially placed targets with either leg in a randomized order was given to the subjects. The average responses of the mediolateral center of pressure (ML-CoP) and center of mass estimate (CoMest) for children with CP and for the typically developing children were later analyzed to observe how the altered anticipatory postural adjustments (APAs) in children with CP affect their dynamic balance (Rapson et al., 2023). In two studies, Ali et al. and Elnaggar et al., performed core stability assessments pre- and post-intervention using the Biodex Balance System to evaluate stability indices, including anteroposterior and mediolateral stability (see Table 2 for more details) (Ali et al., 2019; Elnaggar et al., 2024). It is a multi-axis device that objectively measures a person’s ability to stabilize joints under dynamic loading using a circular platform movable in both the anteroposterior and mediolateral axes and is also suitable for balance training.

As an alternate approach, balance in children with CP can also be studied by measuring the strength of muscles and their coordinated work. A study by Willaert et al. addresses the question of whether CoM feedback can explain reactive muscle activity in children with CP (Willaert et al., 2024). They also considered the possibility that CoM feedback may exhibit higher gain in children with CP compared to children with typical development. Healthcare specialists and researchers look at several key muscle groups during balance analysis, for example, Tibialis anterior (TA) is responsible for raising the foot and controlling the position of the foot in space; Peroneus longus (PL) supports the arch of the foot and is involved in its rotation; Gastrocnemius medialis (MG) and lateralis (LG) plays an important role in raising the toes and stabilizing the ankle joint; Vastus lateralis (VL) is responsible for expanding the leg at the knee joint; Soleus (Sol) plays a key role in maintaining a constant body position in the vertical plane and stabilizing the ankle joint during static and dynamic activity (Han et al., 2024; Willaert et al., 2024). Likewise, Gluteus medius (Gmed) is involved in stabilizing the hip joint and controlling lateral movements of the pelvis, while Gluteus maximus (Gmax) is responsible for hip extension and is also involved in maintaining an upright body posture (Han et al., 2024).

### 3.1. Dynamic balance assessments

Eight studies were devoted to dynamic balance, assessment of movement stability, and correction of posture during movement. Diverse dynamic balance assessment tests are used in clinical practices owing to their simplicity, cost-effectiveness, and relative quantification. One such clinical test is the 6MWT, which is used to measure aerobic capacity and endurance. Performance on this test is significantly associated with physical activity levels in individuals with CP (Cheng et al., 2020; Conner et al., 2021). The test is administered individually using standard instructions. Subjects walk as far as possible within 6 mins on a flat hard surface, which is at least 30 meters long, and subjects turn around at the ends (Giannitsi et al., 2019; Elnaggar et al., 2023). The result is measured in meters, and healthy subjects usually walked an average distance in 6MWT between 400 and 700 meters (Matos Casano and Anjum, 2023; Yang et al., 2023). During the examination, the patient should be allowed to take breaks and rest as needed, and the patient’s heart rate should be continuously monitored using a portable monitor (An et al., 2024). The 10MWT test is an additional useful method for assessing dynamic balance. The 10MWT is also a common tool for assessing gait speed, functional mobility, and vestibular function in people with gait limitations (Peters et al., 2013; Poncumhak et al., 2014; Lang et al., 2016). Participants perform a 10MWT by walking along a 10-meter path three times at their preferred speed. Two sets of timers can be used to record the results in seconds. Time can be measured while the subject is in the middle of a 10-meter section of a 14-meter track, at a comfortable walking speed (Amatachaya et al., 2020; Cheng et al., 2020). Time is normally recorded from the 2-meter to the 12-meter mark to exclude the acceleration and deceleration phases, although the participant continues to move until the end of the track. Specialists can also measure the time required to cover the total distance (10 m), as well as the time required to cover 6 meters (excluding the initial and last 2 meters) (de Baptista et al., 2020). Another tool that can be used to assess dynamic balance is the Functional Walking Test (FWT). It was specifically designed to assess walking-related balance and functional walking abilities in children. This test is an easy-to-use, reliable, and effective measurement technique. It is broken down into 11 points and grouped into five categories: kneeling, standing from kneeling, standing, walking, and climbing stairs. FWT helps evaluate a child’s ability to start, stop, turn, and get into a position during functional walking (Quinn et al., 2011; Elnaggar et al., 2024).

### 3.2. Gait assessment

Eleven studies focused on gait assessment, analyzing walking patterns, endurance, and gait stability in individuals with CP. Commonly used tools included the 6MWT, Timed Up and Down Stairs (TUDS), Theia3D markerless motion capture system, marker-based systems, and the GAITRite System. One of the most reliable gold standards for gait analysis is the GAITRite System, which serves as a portable tool for automatically measuring gait parameters. The GAITRite track, which is part of this system, provides a reliable and powerful method for quantitative gait analysis (Wellmon, 2007; Roche et al., 2018; Vítečková et al., 2020). The track has sensors built into the mat that are activated when a person starts to walk and when mechanical pressure is applied (GAITRite®, n.d.). GAITRite System allows the measurement of temporal parameters (such as stride time, velocity, and single or double support) and spatial parameters, including step length, stride length, and distance (Steinert et al., 2019; Khalil et al., 2023). Spatiotemporal parameters (STP) are a reliable method for measuring gait, as supported by numerous studies in the literature. These parameters describe some aspects of gait, such as speed of movement, step length, surface contact time, and periods of limb movement. The study of STP is of particular importance in the clinical analysis of gait, as it quantifies the efficiency of walking and also identifies any deviations, which can be important for the diagnosis of patients with various neurological or orthopedic disorders. (Gouelle et al., 2018; Herssens et al., 2021).

Nowadays, gait parameters are often studied using advanced 3D gait analysis technologies. In addition, video-based observational gait analysis (VBOGA) tools such as the Edinburgh Visual Gait Score (EVGS) are commonly used in health care and resource-limited settings due to their cost-effectiveness and time-saving features (Yun et al., 2023).

Yun et al. suggest that EVGS may offer a promising method to identify the relationship between selective motor control improvement and gait performance in children with bilateral spastic CP (see Table 2 for more details) (Yun et al., 2023). Warmerdam and co-authors believe that IMUs are a particularly promising tool, especially for evaluating hand movements, since they allow measuring movements in everyday life (Warmerdam et al., 2020). Authors highlight the importance of monitoring movements in real-world conditions, as it is likely to differ significantly from movements performed in the presence of a medical professional. Although IMUs are effective in tracking gait abnormalities, the research published by Caldas et al. suggests that their integration into clinical practice remains limited due to sensor biases and calibration issues (Caldas et al., 2017). The need to stand still for an extended duration while applying sensors and calibrating with the program makes such devices uncomfortable, especially for patients with CP, who have sensory and cognitive limitations.

Video-based gait analysis can be categorized into two types: marker-based and markerless systems. Flux et al. evaluated the human body model (HBM), a marker-based system optimized for real-time biomechanical analysis, in the context of clinical gait assessment in children with CP (Flux et al., 2020). Their objective was to evaluate how gait characteristics measured by the HBM matched those of traditional models in pediatric neuromotor populations. A key advantage of HBM highlighted in this work is its ability to perform real-time biomechanical analysis, which can improve clinical decision-making and patient engagement in the treatment process. However, marker-based systems are prone to human errors when placing markers, the preparation process takes a long time, and accuracy suffers due to soft tissue artifacts, as the movement of skin and muscles does not always reflect the movement of bones (Gao and Zheng, 2008; Camomilla et al., 2017; Schallig et al., 2021). In this regard, markerless motion capture is becoming a promising alternative: it does not require physical contact, minimally depends on training, is convenient for patients, and reduces the influence of the human factor (Perrott et al., 2017; Kanko et al., 2021).

Wishaupt et al. compared gait kinematics obtained with a markerless motion capture system (Theia3D) and a traditional marker-based system (HBM) in both typically developing children and children with CP. The study found that Theia3D consistently measured greater ankle plantar flexion throughout the gait cycle (Wishaupt et al., 2024). This feature can be explained by differences in the models: Theia3D represents the foot as two separate segments, allowing for separate tracking of the foot and toes, while HBM used to measure kinematic gait features treats the foot as a single unit. Haberfehlner et al. conducted an automated video-based method to assess dystonia during resting positions (“lying down” and “sitting”) in individuals with dyskinetic CP, specifically targeting non-ambulatory participants (GMFCS levels IV and V, aged 4–25 years) (Haberfehlner et al., 2023). This research paper used DeepLabCut, an open-source, markerless motion capture tool. This approach allows for an objective assessment of dystonic movements in population groups that are traditionally difficult to assess using conventional methods (Haberfehlner et al., 2023). Although markerless motion capture offers several practical advantages, it also has some limitations. Like marker-based systems, the results remain limited by the number and placement of cameras, and significantly brighter lighting is required (Wade et al., 2022). In addition, the true accuracy of markerless motion capture systems has not yet been fully validated. Most current open-source algorithms were not originally designed for biomechanical analysis, and the datasets used to train them often suffer from inconsistent and inaccurate labeling (Wade et al., 2022).

Rasmussen et al. performed gait analyses recording video and three-dimensional kinematics and kinetics by an eight-camera Vicon T40 system and two force-plates (Rasmussen et al., 2019). The aim of this research was to evaluate whether individual interdisciplinary interventions based on instrumental gait analysis lead to significant improvements in gait and functional performance. The main outcome was the gait deviation index (GDI), which is a confirmed parameter characterizing gait pathology in comparison with the normative data. Although GDI is a widely used objective gait deviation measurement system, it has minor limitations, including reduced sensitivity close to normal gait (Rasmussen et al., 2019).

Children with CP face challenges that affect their motor function, activity level, and participation in daily life. Measurement of muscle activation has significant potential for making clinical decisions regarding effective treatment of these children (Pitto et al., 2020). Understanding the relationship between spasticity, weakness, gross motor function, and the functional consequences of activity limitation is critical in the management of CP (Kim and Park, 2011). Instrumental gait analysis has made significant contributions to this field. Numerous studies have found a correlation between movement disorders and functional outcomes in children with CP (Kang et al., 2017; Flux et al., 2021). EMG is an alternative way to objectively assess functional muscle strength. One of the advantages of EMG is that it is directly attached to muscles and thus records the activity of fundamental motor units, unlike other instruments that measure muscle force derivatives such as joint moments (Piccinini et al., 2011; Van Gestel et al., 2012). Additionally, EMG data can be used to analyze dynamic balance, playing a vital role in diagnosing neurological conditions and monitoring disease progression.

For gait analysis, Strazza et al. studied surface EMG signals from muscles such as the vastus lateralis, rectus femoris, and medial hamstrings during free walking. Their study focused on parameters such as degree of stimulation, onset and end of muscle activation, and frequency of occurrence (Strazza et al., 2017). Similarly, Van Gestel et al. collected surface EMG data from the lateral gastrocnemius and medial hamstrings during three-dimensional gait analysis to assess functional muscle strength in children with CP (Van Gestel et al., 2012). Flux et al. measured muscle strength by placing EMG electrodes on the medial gastrocnemius, soleus, and tibialis anterior muscles to examine stretch reflexes of the gastrocnemius muscles using a treadmill perturbation protocol (Flux et al., 2021). Moreover, knowing that crouch gait is associated with extensor muscle weakness, Kang et al. measured EMG activity in the gastrocnemius and soleus muscles (see Table 2 for more details) (Kang et al., 2017). Selection of a muscle group for analysis depends on the specific objectives of the study and the desired data to be collected.

### 3.3. Functional balance assessments

Six of the selected studies examined the ability of participants with CP to maintain functional balance. Jha et al. highlight the effectiveness of multidimensional clinical tools in assessing functional balance and independence in children with bilateral spastic CP. The integration of PBS, Kids-Mini-Balance Evaluation System Test (Kids-Mini-BESTest), GMFM-88, and Wee-Functional Independence Measure (WeeFIM) provided a comprehensive functional assessment, highlighting the importance of balance training in CP rehabilitation programs (Jha et al., 2021). Functional balance can be assessed through clinical examinations based on observation and manual testing, classified using rating scales, and measured using force platforms to obtain quantitative data (Alonso et al., 2014). One of the commonly used clinical tools for assessing functional balance is the Berg Balance Scale (BBS) or its pediatric equivalent, the PBS, developed specifically for school-age children with mild to moderate movement impairments (Franjoine et al., 2003; Lim, 2015). The BBS is considered the gold standard for clinical assessment of functional balance, assessing both static and dynamic balance during functional movements (Lin et al., 2022). Both the BBS and PBS consist of 14 test items, scored from 0 points (indicating the lowest feature) to 4 points (indicating the highest feature), resulting in a maximum score of 56 points (de AC Duarte et al., 2014; Lin et al., 2022; Mohammed et al., 2023; Rastgar Koutenaei et al., 2023; Stergiou et al., 2024). However, it should be noted that according to Jantakata et al., PBS may be a simpler test for assessing functional balance in adolescents with mild CP compared to BBS (Jantakat et al., 2015).

The TUG test is a reliable and valid tool for assessing functional mobility and dynamic balance in people with CP (Hassani et al., 2014). The TUG test is known for its effectiveness, accuracy, and practicality, making it suitable for both adults and children. This test provides quick measurements of gait speed, balance, and overall functional mobility. The purpose of the TUG test is to measure the time in seconds that takes a person to complete a task (Nicolini-Panisson and Donadio, 2013; Alonso et al., 2014). Several studies have shown that the TUG test is particularly effective in differentiating between people who are prone to falls and those who are not, especially in populations with lower limb disabilities (Dunaway et al., 2014). During the test, participants begin in a seating position on a chair with no backrest and no armrests. At the timer signal, the person should stand up, walk 3 meters, turn 180 degrees, return to the starting point, and then sit down again (Carey et al., 2016; Kara et al., 2019; Bilek and Tekin, 2021). The TUDS can be used to assess functional mobility in children and adolescents with CP at GMFCS level I or II (Elnaggar et al., 2023; Elnaggar et al., 2024). This test evaluates the ability to go up and down stairs while assessing lower limb and trunk strength, coordination, and dynamic balance (Del Corral et al., 2021).

If patients or study participants have already achieved peak performance on other tests such as the BBS and TUGT, the Community Balance and Mobility Scale (CB&M) can be used to assess balance because this scale presents more complex tasks (Elnaggar et al., 2024). The CB&M test includes 13 tasks, six of which require performance on both the affected and unaffected sides. Each task is scored on a scale of 0 to 5, with 0 being unable to complete the task and 5 being successful completion of the task, and the total score ranges from 0 to 96 (Howe et al., 2006; Wright et al., 2010; Balasubramanian, 2015).

The Trunk Management Measurement Scale (TCMS) is an assessment tool that measures static and dynamic trunk control (Severijns et al., 2019; Bilek and Tekin, 2021; López-Ruiz et al., 2023). It provides qualitative information about functioning in three planes: transverse (rotation), frontal (leaning), and sagittal (flexion extension) (Heyrman et al., 2011). The specific movements of dynamic sitting movements such as flexion, extension, lateral flexion, and rotation are used to assess balance (Ozal et al., 2019). The TCMS consists of 15 items and is divided into three subscales: static sitting balance, selective movement control, and dynamic reaching (Heyrman et al., 2011). It has two main components: (a) the torso as a stable support and (b) the torso as an actively moving segment of the body (Mitteregger et al., 2015). The total score of these three subscales represents the TCMS score, which can reach a maximum of 58 points (Rastgar Koutenaei et al., 2023).

### 3.4. Motor function assessment

Motor functions in children with CP are assessed using the GMFCS (Dussault-Picard et al., 2022; Mitchell et al., 2023; Colborne, 2024), Fine Motor Function Assessment (Box and Block Test, Nine-Hole Peg Test) (Gehringer et al., 2023; Hoşbaş and Sertel, 2023), Functional Mobility Scale (FMS) (Fonvig et al., 2024), and 3D motion analysis (Sardoğan et al., 2021; Cacioppo et al., 2022). The GMFM is a universal clinical tool that can be used to assess balance, gait, or motor function, depending on the specific parameters being analyzed. However, Gross Motor Function Measure (GMFM‐66 and GMFM‐88) is the most commonly used approach among others (Pierce et al., 2021). Further, since the GMFM-66 ratings are based on barefoot testing, the GMFM-88 should be used if children are using ambulatory appliances, orthoses, or shoes (further details are in Table 4). Later versions of original GMFM-88 includes GMFM-66, GMFM-66 Basal & Ceiling (GMFM-66-B&C), and GMFM-66 Item Sets (GMFM-66-IS) (Duran et al., 2019; Rivera-Rujana et al., 2022). The GMFCS has been used in several studies examining postural control in children with CP. The following five parameters are used to group GMFM elements: Dimension A for lying and rolling; Dimension B measures sitting; Dimension C for crawling and kneeling; Dimension D for standing; Dimension E assesses walking, running, and jumping (Rivera-Rujana et al., 2022). The five levels of classification are based on self-initiated movements and represent differences in motor performance (Gan et al., 2008). However, in GMFM, only one targeted dimension out of five can be analyzed, as demonstrated in the study conducted by Chinniah et al. They specifically assessed sitting motor function using GMFM-88 (sitting dimension – B) before and after intervention in 30 children with spastic CP (see Table 2 for more details) (Chinniah et al., 2020). Another example is a study conducted by Kaya Kara et al., in which gross motor function was assessed using only two dimensions D (standing) and E (walking, running, and jumping) from the extended and revised version of the GMFM-88 E&R (further details in Table 2) (Palisano et al., 2008; Kara et al., 2019).

**Table 4.** Characteristics of the gross motor function measure

Characteristics	GMFCS-66	GMFCS-88
Target group	Only for children with CP	Can be used with all individuals
Items	66	88
Dimensions	Items identified through Rasch analysis have a unidimensional scale providing interval scaling	five dimensions: A. lying and rolling, B. sitting, C. crawling, E. walking, running, and jumping
Scoring	A free computer program, the Gross Motor Ability Estimator, is required to calculate total scores	Item scores can be summed to calculate raw and percent scores using the formula

Additionally, for dimensions D and E of GMFC that assess standing, walking, running, and jumping, the Quality Functional Measure (QFM) could be incorporated, which evaluates five attributes: alignment, coordination, dissociated movement, stability, and weight-shift (Wright et al., 2014; Rapson et al., 2023). QFM provides a comprehensive assessment of functional performance, offering a more in-depth analysis of a person’s motor abilities and areas that may require intervention or support.

The Barthel Index (BI), an ordinal scale, assesses a person’s ability to perform activities of daily living (ADLs), which are important for assessing motor functions because they are directly correlated (An et al., 2024). The BI consists of ten items: feeding, grooming, bathing, transfers (bed-to-chair-and-back), dressing, bowel, toilet use, bladder, mobility on level surfaces, and stair negotiation (Liu et al., 2020). Nowadays, there are three versions and two modifications of the original Barthel Index: The Collin and the Shah versions. However, this test requires significantly longer time, typically between 24 and 48 hours, to complete.

## 4. Discussion and conclusion

Cerebral palsy encompasses a spectrum of neurological disorders emerging postnatally or in early childhood, impacting muscle coordination, balance, and body movements across their lifespan. Globally, researchers and clinicians are putting much effort into advancing balance and gait training methods for children with CP, aiming to optimize adaptation and enhance daily living activities. However, it is crucial in research not only to innovate effective training methods for balance and gait stability but also to define precise criteria for assessing disabilities and evaluating treatment outcomes. Such criteria are fundamental for accurately assessing technique effectiveness and determining their practical applicability. This study reviewed and identified the state-of-the-art balance assessment methods and their implementation in children with CP. Further details on the assessed studies and which balance tools they utilized are presented in Table 2.

The key factor in choosing the appropriate assessment tool is its validity and reliability in the population of patients with CP. Many clinical tools such as the PBS, measurement of general motor function, and the 6MWT demonstrated high reliability and validity in assessing balance, gait, and motor function in children with CP. However, their subjectivity and dependence on the specialist’s experience may reduce the accuracy in detecting minor changes over time. In contrast, instrumental assessment methods provide objective quantitative data, but their availability, cost, and standardization pose certain challenges. The GAITRite system is widely used and has proven to be a reliable tool for analyzing spatiotemporal gait parameters, while motion capture systems (e.g., Vicon, Qualisys) provide detailed kinematic data, but are less commonly used in clinical practice due to the high cost and complexity of operation. The summary Table 5 is presented, in which the most commonly used tools are classified according to the degree of validation, reflecting which methods are fully validated for patients with CP, and which require further research to confirm their reliability and clinical significance.

**Table 5.** Clinical and instrumental assessment tools by validation status in CP (from selected studies)

Assessment type	Tools	Validated in CP	Outcome measures
Clinical balance assessment	Pediatric Balance Scale (PBS) (Hsieh, 2020; Stergiou et al., 2024; An et al., 2024; Jha et al., 2021; Mohammed et al., 2023; Khalil et al., 2023; Bilek and Tekin, 2021; Rastgar Koutenaei et al., 2023; Yun et al., 2023)	Yes	Postural stability, static/dynamic balance
	Gross Motor Function Measure (GMFM) (Cho and Lee, 2020; Rapson et al., 2023; Stergiou et al., 2024; Jha et al., 2021; Kara et al., 2019; Rastgar Koutenaei et al., 2023; Chinniah et al., 2020; Fu et al., 2022; Yun et al., 2023)	Yes	Functional balance, motor function
	Timed Up and Down Stair Test (TUDS) (Elnaggar et al., 2024, 2023)	Yes	Dynamic balance, mobility
	Community Balance and Mobility Scale (CB&M) (Elnaggar et al., 2024)	Limited	High-functioning CP balance
	The Trunk Control Measurement Scale (TCMS) (Rastgar Koutenaei et al., 2023)	Limited	Trunk control in 3 dimensions
Instrumental balance assessment	Biodex Balance System (Ali et al., 2019; Elnaggar et al., 2024; 2023)	Yes	Postural control, sway analysis
	Force Plates (Rapson et al., 2023; Hsieh, 2020)	Yes	Center of Pressure (COP), postural sway
Clinical gait assessment	Six-Minute Walk Test (6MWT) (Elnaggar et al., 2023; An et al., 2024; Fu et al., 2022)	Yes	Endurance, walking ability
	Functional Ambulation Category (FAC) (An et al., 2024)	Yes	Ambulation independence
	Timed Up and Go Test (TUGT) (Bilek and Tekin, 2021; Kara et al., 2019; Yun et al., 2023)	Yes	Dynamic balance, mobility
	Ten-Meter Walk Test (10MWT) (Yun et al., 2023)	Yes	Walking speed, stride length
Instrumental gait assessment	GAITRite System (Elnaggar et al., 2023; Khalil et al., 2023)	Yes	Spatiotemporal gait parameters
	Motion Capture (Hegazy et al., 2020; Rasmussen et al., 2019) (markerless (Wishaupt et al., 2024) and marker-based (Flux et al., 2020))	Limited	Joint kinematics, gait biomechanics
	Electromyography (EMG) (Fu et al., 2022; Hegazy et al., 2020)	Yes	Muscle activation, gait efficiency

PBS was the most frequently used clinical tool, featured in nine studies (Hsieh, 2020; Bilek and Tekin, 2021; Jha et al., 2021; Khalil et al., 2023; Mohammed et al., 2023; Rastgar Koutenaei et al., 2023; Yun et al., 2023; An et al., 2024; Stergiou et al., 2024). As we mentioned before, PBS is a pediatric version of the BBS which is considered the gold standard. Comprehensive assessment of functional balance in children makes PBS a reliable choice for both clinical and research purposes. The Gross Motor Function in its original (GMFM-88) and later versions were also mentioned in nine articles, emphasizing its importance in assessing gross motor skills in children with CP according to Table 2 (Kara et al., 2019; Chinniah et al., 2020; Cho and Lee, 2020; Jha et al., 2021; Fu et al., 2022; Rapson et al., 2023; Rastgar Koutenaei et al., 2023; Yun et al., 2023; Stergiou et al., 2024). These tools are essential for understanding balance and gait stability in daily activities and overall mobility. However, they are subjective, and individual specialists may come up with different scores using these tools. On the other hand, instrument-based assessments provide quantitative data for more precise measurements of muscle activations, CoM, CoP, gait STP, and so forth. The Biodex Balance System was used in three studies to evaluate postural balance (Ali et al., 2019; Elnaggar et al., 2023; Elnaggar et al., 2024). The Biodex Balance System measures tilt around each axis under dynamic conditions and calculates the anterior–posterior stability index (APSI), medial-lateral stability index (MLSI), and overall stability index (OSI), which are considered the best indicators of a patient’s ability to maintain balance. The GAITRite system and portable force plates have also been used in numerous studies to evaluate gait and balance parameters. Various gait indices proposed by the researchers are being used to assess overall gait pathology and outcomes from interventions using instrumented gait analyses. Although they are not popular among CP trials, several indices have been proposed in the literature to quantify gait disorders. These include Gait Posture index (GPi) (Mobbs et al., 2019), Gait Abnormality Index (GAI) (Langley and Greig, 2023), Gillette Gait Index (GGI), Gait Deviation Index (GDI), Gait Profile Score (GPS), and Gait Quality Index (GQI) (Kingsbury et al., 2017). These tools allow an objective analysis of gait deviations based on kinematic and kinetic parameters, providing valuable information about motor disorders. Despite their limited use in CP research, they have the potential to improve the accuracy of gait assessment and clinical decision-making. Researchers have also proposed a few combined gait asymmetry metric (CGAM) by merging spatial, temporal, kinematic, and kinetic gait parameters and later comparing it with the clinical measures (Ramakrishnan et al., 2019). Marker-based and markerless motion capture systems have various advantages and limitations in the clinical analysis of gait. Marker-based systems, such as the HBM, are widely considered the gold standard due to their high accuracy and validation in a variety of clinical settings (Van den Bogert et al., 2013; Wishaupt et al., 2024). However, these systems are time-consuming, require qualified personnel, and are prone to soft tissue artefacts and calibration errors, especially in children with increased motor impairments (Wishaupt et al., 2024; Poomulna et al., 2025). In contrast, markerless systems, such as approaches based on 3D or 2D video, eliminate the need for physical markers, reduce setup time, and are more convenient and accessible (Pantzar-Castilla et al., 2018; Kanko et al., 2021; Wade et al., 2022; Lam et al., 2023). However, their accuracy and sensitivity, especially when detecting minor deviations in gait, remain at the development stage (Wade et al., 2022). Differences in segment definitions and postural assessment models can affect derived metrics such as the Gait Deviation Index, as can be seen from recent studies showing consistently lower GDI scores from Theia3D compared to marker-based systems (Poomulna et al., 2025). IMUs and EMG are widely used due to their ability to provide real-time data and accurate measurement. IMUs provide precise measurements of the movement dynamics (accelerations, anatomical joint angles, and orientation angles), whereas EMGs provide detailed information about muscle activity and help to detect neuromuscular abnormalities. The wide range of assessment tools highlights the complexity of balance disorders in children with CP and the need for an integrated approach to assessing balance and gait stability. Currently, there is no consensus on preferred assessment instruments; selection should be customized to the specific intervention goals and expected results. The summarized tools from the current study will be able to assist clinicians in choosing proper balance assessment tools.

Looking forward, there is a need to devise new metrics combining clinical and instrumental (EMG, IMU, etc.) measures. Artificial intelligence (AI) may be a possible tool to combine evidence-based evaluations of CP children. For example, these metrics may combine balance parameters, taking into account both static and dynamic components for better balance profiling. Moreover, such indices may capture the nuanced differences between children at the same GMFCS level and may be useful in monitoring progress dynamically. These indices may also help in creating individualized profiles that reflect the unique motor abilities and cognition of each child and enable clinicians to design and implement more targeted and personalized intervention strategies.

AI models could be trained to process a large amount of biomechanical information, detecting minor deviations in movement that might be missed during clinical examination. There are several Machine Learning Algorithms such as K-Star, multilayer perceptron (MLP), naïve Bayes (NB), random tree (RT), and support vector machine (SVM) that have been used to predict CP and classify gait patterns in CP patients (Balgude et al., 2024), as well as K-means clustering and Principal Component Analysis (PCA) that are effective in identifying novel gait patterns and subgrouping CP severity levels (Katmah et al., 2023). Balgude et al. conducted extensive research and listed some machine learning and deep learning models used in CP patient care research, citing their advantages and disadvantages (Balgude et al., 2024). Machine learning technology is also being used in robot-assisted therapy, which makes it possible to increase the effectiveness of gait correction by constantly adjusting interventions depending on the patient’s treatment results. (Mehr et al., 2023). Shefa et al. proposed a machine learning approach for managing gait issues using an Ankle-foot orthosis (AFO) equipped with surface EMG sensors and an IMU to monitor muscle activity and gait movements, allowing accurate prediction and evaluation of patient’s walking patterns (Shefa et al., 2024). Computer vision and deep learning models provide increased visibility in gait analysis and motion capture during home-based rehabilitation programs by extracting key kinematic joint characteristics and spatiotemporal gait characteristics from images and videos (Sardari et al., 2023).

Most AI-based gait analysis tools have been trained on healthy individuals or small, homogeneous CP populations, limiting their generalizability. To mitigate bias, training datasets must include diverse CP cases across different age groups, motor severities, and intervention histories. In AI research on CP, several datasets are commonly used to analyze gait and motion, including MINI RGBD with 12 subjects aged 0–7 months, which was used for the early prediction of CP by McCay et al. (2021), Wu et al. (2023), and Devarajan and Khader (2023). The RVI-38 dataset is a real patient video dataset collected at the Royal Victoria Infirmary (RVI) with 38 subjects aged 3–5 months (https://github.com/edmondslho/Pose-basedCerebralPalsyPrediction), BabyPose (Migliorelli et al., 2020) with 16 preterm infants and Motion Infant Analysis (MIA) (https://vrai.dii.univpm.it/mia-dataset) with one preterm infant at 37 + 1 weeks gestational age. However, we still need more robust, clinically relevant federated datasets to refine AI-based CP assessment tools.

With ongoing advancements in technology enhancing our capability to gather and interpret biomechanical and neuromuscular data, we expect increased accuracy not only in predicting CP but also in identifying specific types of functional capabilities and movement disorders. By the combination of clinical assessments with instrumental data (EMG, IMU, etc.), researchers and clinicians can identify patterns, biomarkers, or specific parameters that indicate different functional levels. Such integration of objective data with clinical insights can facilitate the development of predictive models or algorithms that may accurately determine GMFCS levels based on instrumental measurements.

Addressing the accessibility and functionality of advanced assessment tools is also a major focus. The development of cost-effective and user-friendly instruments may facilitate their broad support in a variety of health care and research centers, including resource-limited settings, which in turn would help standardize the measurement of balance and gait stability.

## Data Availability

All data and material reported in this systematic review are from peer-reviewed publications.

## Abbreviations

10mSRT: Ten-Meter Shuttle Run Test
10MWT: Ten-Meter Walk Test
6 MWT: Six-Minute Walk Test
BBS: Berg Balance Scale
BESS: Balance Error Scoring System
BoS: Base of Support
CB&M: Community Balance and Mobility Scale
FAC: Functional Ambulation Category
FRT: Functional Reach Test
FTSST: Five Times Sit-to-Stand Test
FWT: Functional Walking Test
GMFCS: Gross Motor Function Classification System
MBI: Modified Barthel Index
MCSI: Modified Caregiver Strain Index
PBS: Pediatric Balance Scale
QFM: Quality Functional Measure
TCMS: Trunk Control Measurement Scale
TIS: Trunk Impairment Scale
TUDT: Timed Up and Down Stairs Test
TUGT: Timed Up and Go Test
WeeFIM: Wee-Functional Independence Measure
